# Fabrication and Characterization of Hydrogels Based on Gelatinised Collagen with Potential Application in Tissue Engineering

**DOI:** 10.3390/polym12051146

**Published:** 2020-05-17

**Authors:** Victor Perez-Puyana, Mercedes Jiménez-Rosado, Alberto Romero, Antonio Guerrero

**Affiliations:** 1 Departamento de Ingeniería Química, Facultad de Química, Universidad de Sevilla, 41012 Sevilla, Spain; vperez11@us.es (V.P.-P.); alromero@us.es (A.R.); 2 Departamento de Ingeniería Química, Escuela Politécnica Superior, Universidad de Sevilla, 41011 Sevilla, Spain; mjimenez42@us.es

**Keywords:** gelatinised collagen, hydrogel, rheology, Cryo-SEM, tissue engineering

## Abstract

Regenerative medicine is increasingly focused on the development of biomaterials that facilitate cell adhesion and proliferation through the use of natural polymers, which have better biocompatibility and biodegradability. In this way, the use of hydrogels has been considered as a potential option for tissue engineering due to their physical and chemical characteristics. However, few studies associate the raw materials properties and processing conditions with the final characteristics of hydrogels, which could condition their use as scaffolds for tissue engineering. In this context, the main objective of this work was the evaluation of type I collagen as raw material for the elaboration of hydrogels. In addition, gelation time, pH and temperature were evaluated as the most influential variables in the hydrogel processing method by rheological (time, strain and frequency sweep tests) and microstructural (Cryo-SEM) measurements. The results indicate that it is possible to obtain collagen hydrogels with adequate rheological and microstructural characteristics by selecting optimal processing conditions. However, further studies are necessary to assess their suitability for cell accommodation and growth.

## 1. Introduction

Regenerative medicine has long been just an unattainable dream; however, with the recent technological advancements and the efforts of the scientific community, this branch of medicine is currently opening the way to the future, developing treatments against many diseases. This medical science works alongside with others, such as advanced cell therapy, genetic engineering and tissue engineering, which are the main fields that can lead to the accomplishment of the objectives proposed by this branch of medicine [1]. Tissue engineering is defined as “the science of designing and manufacturing new tissues for the functional restoration of altered organs and the replacement of structures damaged by trauma or disease” [2]. Hence, it is a growing multidisciplinary field that implies different areas, such as chemistry, biology, engineering and medicine. As a consequence of the expansion of this research field, a new type of materials was developed, called biomaterials. Among the multiple definitions given to biomaterials over the years, the most generally accepted one outlines the term as “a material designed to repair or restore the functionalities of a defective biological system” [3].

As a historical review, the first articles written about these materials date from the late 1960s and early 1970s [4,5]. From these years, the analysis of these materials has been one of the most studied topics due to their versatility. Thus, biomaterials have evolved throughout history, improving their characteristics. Among the target properties, they must be biocompatible and non-toxic, and their macroscopic structure must have the characteristics of the material to be replaced (meeting their biomechanical, biofunctional and biomimetic needs). Furthermore, they must be stable during sterilization and storage, as well as during implantation. Depending on the application, they can be permanent or temporary (in this case, their degradation rate should be similar to the regeneration rate) [6,7].

The development of a biomaterial encompasses three different stages: the selection of the raw material, the processing technique and the characterization of the final biomaterial. First of all, the selection of the raw material to use is often based on the processing technique and the properties that can be benefited from them [8]. It is preferable to use raw materials that allow a better control of the properties of the final biomaterials. However, those materials that best allow controlling their properties often have the disadvantage of not being biocompatible or having toxic degradation or corrosive products. In this way, bioinert materials (first generation biomaterials) stand out for their lack of interaction with the organism. Titanium and polymethyl methacrylate can be highlighted as examples of this type of materials [9,10]. However, biomaterials with other properties are currently being sought, more related to facilitating cell adhesion and proliferation (third generation biomaterials) [11,12]. Most of the studies that are currently being carried out are based on the use of natural polymers, due to their excellent biological properties (biocompatibility and biodegradability) [13]. Thus, although the mechanical properties need to be reinforced, these polymers provide better cell adhesion and interaction with the host tissue [14]. From all the possible biopolymers, collagen can be highlighted as a versatile biopolymer that has been used to produce biomaterials with several different techniques, granting different final characteristics to the biomaterial [15,16,17,18,19]. Collagen-based scaffolds may be constructed either in the native form or in the denatured form (gelatin). Gelatin, the natural polymer obtained from collagen by acidic or alkaline denaturation, is widely used due to its advantages when used as a raw material in tissue engineering applications. However, the denaturation process is not often complete, and, in most cases, the product obtained is a gelatinised collagen protein rather than pure gelatin protein.

Several processing techniques have been used to develop three-dimensional biomaterials, such as 3D printing, electrospinning and freeze-drying [20,21,22,23,24]. Among them, the formation of hydrogels is widely used for the treatment of materials produced with natural polymers [25]. A hydrogel is a three-dimensional cross-linked polymer network that can absorb and hold large amounts of water in the interstitial spaces between the polymer chains [26]. There are different classifications of hydrogels according to the configuration, composition, source or the type of crosslinking. The latter divides hydrogels in chemically or physically induced hydrogels, depending on if the junctions are based on chemical networks or physical interactions [27]. Apart from that, there are other hydrogels, the so-called stimuli-responsive hydrogels, which respond to changes in the external environment [28]. These stimuli provoke swelling of the structure that could be so drastic to produce a phase transition [29].

An ideal hydrogel must present specific functional features such as a high absorption capacity, low price, high durability and stability, colourlessness, good biodegradability, etc. [30]. Depending on the properties exhibited by the hydrogels, they can be used in several applications. Apart from regenerative medicine, these hydrogel materials may be applied to hygienic products [31], drug delivery systems [32], biosensors [33], etc. Among the different raw materials to be used, natural polymers are the most studied due to the final properties of the scaffolds [34,35]. Proteins as collagen or keratin are promising options as shown in studies driven by Peppas and Khare (1993) or Tachibana et al. (2002), in which those proteins have been used to produce hydrogels with potential applicability in drug delivery or wound healing applications, respectively [36,37]. On the other hand, polysaccharides as chitosan or alginate have been extensively used as raw materials for the fabrication of hydrogels. For example, the studies of Malafaya et al. (2005), where chitosan was used to produce scaffolds for osteochondral tissue engineering or the work of Augst et al. (2006) about alginate hydrogels [38,39].

In general, those studies have produced hydrogels following specific conditions. Therefore, an important stage during the fabrication of materials is the choice of the most suitable processing conditions in order to obtain structures with desirable properties. Regarding the modification of the processing variables, the different parameters to be considered in the fabrication process of hydrogels are gelation time, pH value of the solution, and gelation temperature. Among these, depending on the parameter to be modified, pH-sensitive or temperature-sensitive hydrogels can be found [28].

Due to their chemical and physical characteristics, hydrogels have been presented as potential candidates in pharmaceutical and biomedical applications [27]. In this sense, it is important to characterise the mechanical and stability properties of the hydrogels, as well as their morphology, to ensure a good comprehension of their structure. The former can be evaluated through rheological measurements in order to analyse the stability and mechanical strength of hydrogels. On the other hand, the morphology can be assessed with microscopy analyses to properly visualise the internal and surface structure of the hydrogel.

As mentioned above, besides characterising the properties of the final structures formed, it is also important to properly select and characterise the raw material before proceeding to the fabrication of the hydrogel, since, apart from the mechanical properties shown, they should be built using materials that allow cells to maintain their phenotype [40]. Nevertheless, only a few studies are based on the evaluation of the properties of the raw material to be used together with the assessment of the processing parameters in the properties of the final biomaterials.

Apart from measuring the mechanical properties of the hydrogels, rheology can also be used to analyse the gelation process of these systems by measuring the response of the hydrogels to an applied constant stress over time. Interestingly, the main novelty of this work is based on the evaluation of the changes in the structure of the hydrogel via a rheological analysis through the control of the gelation process in situ. In addition, the influence of the different processing parameters (gelation time, pH, and gelation temperature) on the properties of collagen-based hydrogels was also studied.

Therefore, the objective of this research was the use of rheology as a tool to evaluate the gelation process of gelatinised collagen-based hydrogels. In addition, the optimization of the different processing variables involved in the formation of hydrogels was also carried out. To this end, the rheological and morphological properties of the hydrogels based on gelatinised collagen were evaluated. Furthermore, an additional objective of this work was the characterization of the raw material to be used for the fabrication of hydrogels.

## 2. Material and Methods

### 2.1. Materials

Collagen protein from Essentia Protein Solutions (Graasten, Denmark) was used in this study. The technical data sheet of the product indicates that it is type I collagen obtained from pork, with a percentage of protein matter greater than 90 wt%; no further specific data on its composition was provided. This collagen protein presented a denaturation degree of 75% [41], being considered as a gelatinised collagen protein, rather than a pure collagen protein, in the further sections. Moreover, the amino acid profile is similar to the profile observed in other collagen proteins, revealing a high content in glycine, glutamic acid, alanine and arginine [42]. In addition, acetic acid supplied by PANREAC (Barcelona, Spain) was used as a solvent.

### 2.2. Characterization of the Raw Material

#### 2.2.1. Analysis of the Chemical Composition

The protein content of the gelatinised collagen was determined using a LECO CHNS-932 microanalyzer (Leco Corporation, St. Joseph, MI, USA) to measure the nitrogen content of the sample and multiplying this by 5.95 [43]. Likewise, the lipid, moisture and ash contents were determined according to A.O.A.C. protocols [44]. The Soxhlet method was used to quantify the lipid content. In the present study, it consisted in heating and volatilizing a solvent (hexane) at 80 °C and, subsequently, condensing the solvent to bring it into contact with the sample in a Soxhlet extractor, allowing 4 h for the solvent to leach the lipids out of the sample. The ash content was obtained by calcining 2.0 g of protein at 550 °C in a muffle furnace (ST Tecnylab, Valencia, Spain) for 4–5 h and then weighing the calcined sample after cooling in a desiccant at room temperature (22 ± 2 °C). In addition, the moisture of the sample was determined by treating a 1.5−2.0 g sample in a furnace (Incubat, Selecta, Valencia, Spain) at 105 °C for 24 h. The lipid, moisture and ash contents were calculated using the following equation (Equation (1)):

(1) $$\mathrm{wt}\;\%\;\mathrm{Component}=\frac{\mathrm{Initial}\;\mathrm{sample}\;\mathrm{weight}\;\left(g\right)-\mathrm{Final}\;\mathrm{sample}\;\mathrm{weight}\left(g\right)}{\mathrm{Initial}\;\mathrm{sample}\;\mathrm{weight}\;\left(g\right)}\times 100$$

#### 2.2.2. Protein Solubility and Z-Potential

Protein solubility was determined at different pH values. Aqueous dispersions (ca 0.025 g/mL) were prepared with buffers at different pH values. The samples were homogenised and subsequently centrifuged for 20 min at 15,000 rpm and 10 °C. The supernatants were collected for protein content measurement by the Markwell method [45]. Solubility was expressed as a percentage (g soluble protein/100 g isolate in the sample).

Together with the solubility over the pH range, the isoelectric point was measured using a “Zetasizer 2000” (Malvern Instruments, Malvern, UK). Different samples were prepared at 1 wt % with buffers at different pH values. Prior to analysis, the samples were stirred at 20 °C and then centrifuged at 12,000 rpm for 10 min in a RC5C Sorvall centrifuge (Sorvall Instruments, Waltham, MA, USA). After that, the samples were measured in triplicate at 20 °C. The zeta potential was calculated from electrophoretic mobility using the Henry equation and the Smoluchowski approximation. The isoelectric point corresponds to the point at which the potential value is zero, due to the neutralisation of the surface charge of particles [46].

#### 2.2.3. Fourier Transform Infrared Spectroscopy (FTIR)

The chemical bonds were analysed by the FTIR method using an FTIR-4100 spectrophotometer (Jasco, Tokyo, Japan). The collagen samples were introduced in the equipment and subjected to modulated mid-infrared energy, which was absorbed at specific frequencies. These absorptions are related to the vibrational bond energies of the functional groups present in collagen [47]. Thus, the different spectra were collected in the range of 4000-600 cm^−1^.

### 2.3. Formation of Hydrogels

The scaffolds were processed by a gelation process to fabricate hydrogels. It is important to point out that the process is similar to other studies concerning protein-based hydrogels [48]. The hydrogels were produced from a collagen solution (10 mg/mL) in 0.05 M acetic acid, which was centrifuged at 12,000 rpm, maintaining the temperature of the solution at 4 °C. The selection of the pH and acid used was based on a previous study, in which this combination exhibited the best results without the modification of the structure of the protein [41]. Subsequently, a gelation step took place under specific conditions to evaluate the influence of the different processing parameters on the properties of the hydrogels. Therefore, the gelation time (1, 2 and 4 h), the pH (3, 5, 6.5, 8 and 10) and the gelation temperature (4 and 20 °C) were evaluated.

### 2.4. Characterization of the Hydrogels

Once the hydrogels were produced, they were characterised by a rheological study and the evaluation of their morphological properties.

#### 2.4.1. Rheological Evaluation

The viscoelastic properties of the hydrogels were determined by using three types of rheological tests carried out with an AR 2000 rheometer (TA Instruments, New Castle, DE, USA):

- Strain sweep tests: Measurements between 0.1% and 100% strain and a constant frequency of 1 Hz were performed to determine the linear viscoelastic range (interval where the elastic and viscous moduli are independent of the strain) and the critical strain (the maximum strain supported by the sample within the linear viscoelastic range). These tests were performed at 37 °C to simulate the potential behaviour of the hydrogels in the body.
- Frequency sweep tests: The measurements were carried out in a frequency range between 0.1 and 10 Hz at a specific strain for each system (within the linear viscoelastic range). In these tests, the elastic and viscous moduli (G’ and G’’, respectively) were obtained, together with the loss tangent (tan δ) and complex viscosity (η*). In a similar way than the strain sweep tests, these measurements were performed at 37 °C to simulate the potential stability of the hydrogels in the body.
- Time sweep tests: In this case, the test was carried out to evaluate the gelation process of the hydrogels. The test was performed at constant frequency (1 Hz), strain (3%) and temperature (4 °C) for a certain time (4 h) under the gelation conditions used.

All these tests were carried out with a serrated plate-plate geometry (diameter: 40 mm) and controlling the temperature with a Peltier connected to a thermostatic bath.

#### 2.4.2. Morphological Evaluation 

Morphological examination of hydrogels was assessed with a cryo-scanning electron microscope (Cryo-SEM) and a Zeiss EVO (Jena, Germany) at an acceleration voltage of 10 kV. The samples were previously cooled with liquid nitrogen and covered with a film of Au in a high-resolution sputter coater Leica (Wetzlar, Germany).. A digital processing software, ImageJ (National Institutes of Health, Bethesda, MD, USA), was used to determine the pore size distribution and the mean pore size of the selected hydrogels. 

### 2.5. Statistical Analysis

At least three replicates were carried out for each measurement. Statistical analyses were performed with *t*-tests and a one-way analysis of variance (*p* < 0.05) using PASW Statistics for Windows (Version 18: SPSS, Chicago, IL, USA). Standard deviations were calculated for selected parameters.

## 3. Results and Discussion

### 3.1. Characterization of the Raw Material

The chemical composition analysis of the raw material showed a high protein content (ca. 91 wt %), indicating that, according to Pearson’s classification, it can be considered as a protein isolate [49]. On the other hand, low percentages of lipids and ash were found (0.7 and 0.8 wt %, respectively). These results are similar to those obtained by Liang et al. (2014) [43], although with a slightly higher content in ash and lipids.

**Figure 1:**
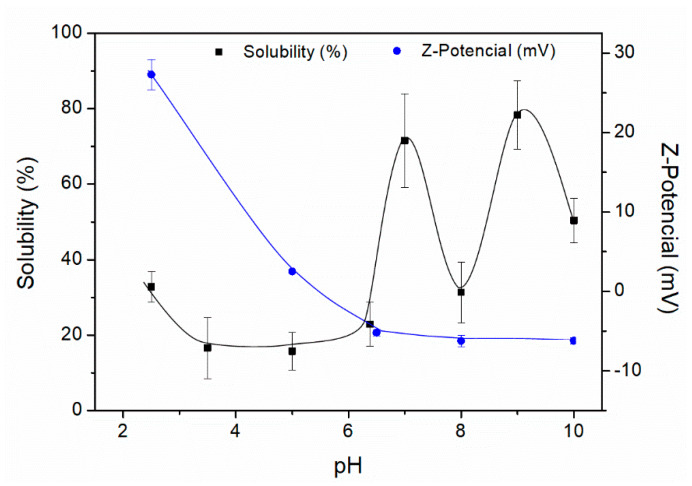
Protein solubility (%) and Z-Potential (mV) of the collagen protein isolate.

Figure 1 shows the percentage of protein solubility, as well as the Z-Potential values as a function of pH for the gelatinised collagen protein isolate. The solubility is high at basic pH values, reaching maximum values (ca. 80%) at pH 9. The obtained profile shows a sudden rise in solubility from pH 4 to 7 and a sudden decay at pH 8. The isoelectric point of this protein, the pH at which the total net charge is 0, is between 5 and 6 according to the profile exhibited. The obtained profile differed from the one obtained by Veeruraj et al. (2013) [50], although the range found for the isoelectric point is similar. A better approach to the isoelectric point of the protein isolate was obtained by Z-Potential measurements (Figure 1). The Z-Potential values were studied at different pH values, passing from positive to negative values (from 27 mV at pH 2.5 to −6 mV at pH 10). The pH at which the Z-Potential is zero corresponded to the isoelectric point, being in the range of pH 5–6 (pH 5.2, approximately). The results revealed a good correlation between the solubility and Z-Potential values.

**Figure 2:**
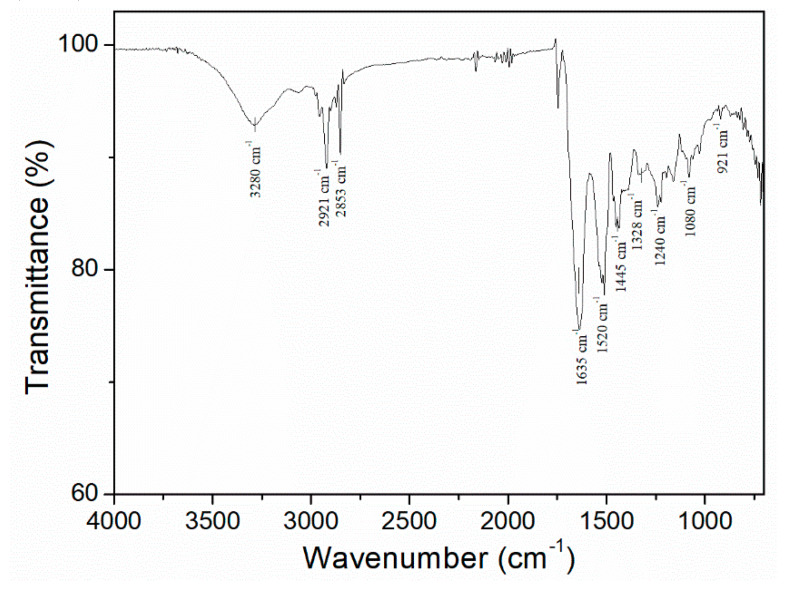
FTIR profile of the collagen protein isolate.

Finally, the characterization of the raw material encompassed the analysis via infrared spectroscopy (Figure 2). The spectrum of this protein isolate presents five typical signals of gelatinised collagen protein: A first signal around 3400–3300 cm^−1^, referring to N–H stretching, followed by another signal at 3000–2900 cm^−1^, characteristic of C–H stretching (CH_2_). Next, three signals appear at 1650, 1550 and 1200 cm^−1^, related to C=O and C–N stretching and N–H bending, respectively. The peaks observed are in the same range as the ones carried out by Muyonga et al. (2004) or Payne et al. (1988) [51,52].

### 3.2. Characterization of Hydrogels 

#### 3.2.1. Influence of the Gelation Time

**Figure 3:**
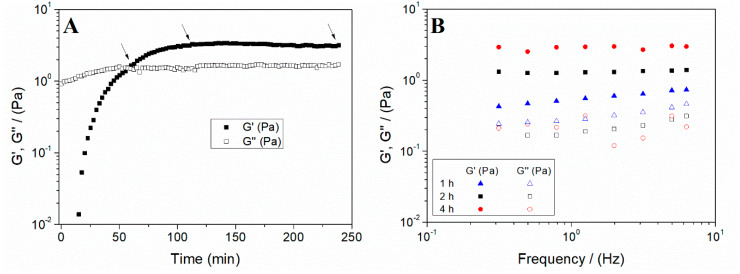
(**A**) Time sweep test of a collagen hydrogel (the arrows included indicate the gelation times selected for the further study) and (**B**) evolution of the elastic and viscous moduli (G’ and G’’, respectively) of the collagen hydrogels processed at different gelation times (1, 2 and 4 h).

The study of the gelation time allows evaluating the optimal time for the formation of hydrogels. For this reason, the first step was to perform a time sweep test to analyse the evolution of the gelation process of the systems (Figure 3A).

The profile shows how a crossover point takes place between G’ and G’’ values after 1 h. Several authors have outlined this crossover as the gelation point. However, although both are close, it is generally recognised that the gel point does not necessarily match the crossover point [53]. Soon after the crossover point, nearly constant values of G’ and G’’ were achieved. Therefore, 1, 2 and 4 h were selected as possible gelation times to check their influence on the properties of hydrogels.

Then, strain sweep tests were carried out at a constant frequency (1 Hz). From the values obtained, stress–strain curves were used (data not shown) to obtain the limit strain values in the linear viscoelastic range (known as critical strain). The critical strain was calculated for each system evaluated and the results obtained are summarised in Table 1. The critical strain values show that the gelation time did not produce significant changes in the deformability of the hydrogels after a gelation time of 2 h.

In addition to the critical strain, frequency sweep tests were performed for the scaffolds processed at different gelation times. The profiles obtained are shown in Figure 3B. It is observed that G’ was much greater than G’’ throughout the studied interval, thus showing a predominantly elastic behaviour. This response is a general feature of gels, found either in protein-based hydrogels [21,53] or in polysaccharide-based hydrogels [54,55]. Furthermore, both the elastic and viscous moduli showed a small dependence on frequency (i.e., G’ remained nearly constant within the frequency range evaluated for the longer gelation times), demonstrating the stability of these systems. This behaviour is typical of hydrogel-based systems [56,57]. To show the significant differences between the systems, the values of G’, tan δ and η* at 1 Hz (G’_1_, tan δ_1_ and η*_1_, respectively) are included in Table 1. These values indicate that a longer gelation time results in a more structured system, i.e., with a higher G’_1_ and η*_1_ value and a lower value of tan δ_1_, although without significant differences after 2 h. Therefore, a gelation time of 2 h was selected for the following studies.

#### 3.2.2. Influence of the pH and Gelation Temperature

The effect of pH (3, 5, 6.5, 8 and 10) was evaluated at two different two gelation temperatures (4 and 20 °C) in this section.

Once again, strain sweep tests were first performed to obtain the critical strain of the different systems. The values are summarised in Table 2. As may be deduced from these values, the hydrogel ability to deform without altering its microstructure, which is reflected by the critical strain value, generally remains constant, except for two cases. An increase in the gelation temperature (from 4 to 20 °C) at low pH (i.e., pH ≤ 6.5) lessens this ability. A minimum value for the critical strain took place at pH 6.5, regardless of the temperature used in the gelation process.

**Figure 4:**
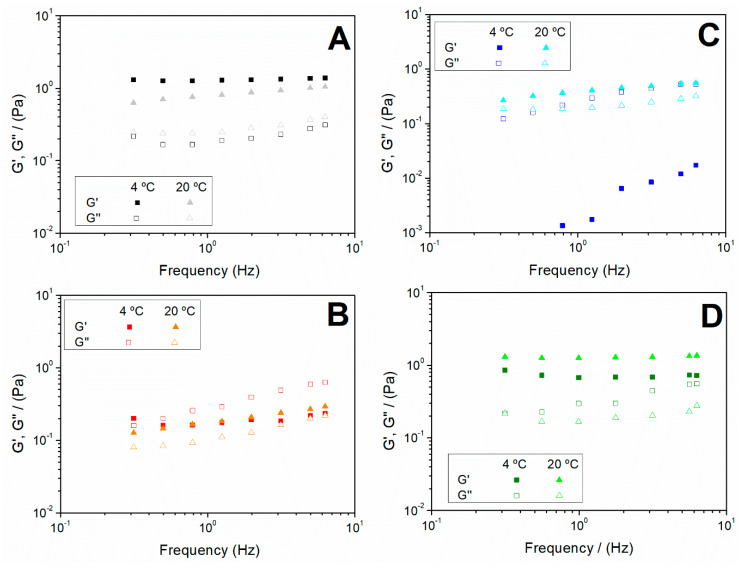
Frequency sweep tests of the hydrogels obtained at 4 and 20 °C and different pH values: (**A**) pH = 3; (**B**) pH = 5; (**C**) pH = 6.5 and (**D**) pH = 10.

In addition, frequency sweep tests were carried out to determine how the pH and the gelation temperature influenced the stability of the systems. Figure 4 shows the frequency sweeps of the systems produced varying the pH values (3, 5, 6.5 and 10) at 4 and 20 °C (Figure 4A–D, respectively). The behaviour found at pH 8 (data not shown) was very similar to that one shown in Figure 4D for pH 10. This similarity can also be inferred from the values reported in Table 2 for both hydrogels. The profiles show an evolution from a more structured system at pH 3 (Figure 4A), with low dependence of both the elastic and viscous moduli on frequency, to systems with a more fluid character when the hydrogel was obtained at a pH between 5 and 7 (Figure 4B,C), which encloses the isoelectric point. In this sense, it should be noted that the viscous modulus becomes more relevant in this pH region (5–7), particularly at low temperature at which the viscous response dominates over the elastic one (G’’ > G’) throughout the frequency range studied. However, a more structured system was again obtained when the hydrogel was processed at basic pH (Figure 4D), with the G’ values being higher than the G’’ values, regardless of the processing temperature. This evolution, together with the decrease in the elastic modulus and the greater dependence of both moduli on frequency, reflect a weakening of the structure in the pH region 5–7.

The values of the elastic modulus (G’), loss tangent (tan δ) and complex viscosity (η*) at 1 Hz (G’_1_, η*_1_ and tan δ_1_, respectively) are summarised in Table 2. A similar evolution to that described for Figure 4 can be observed when comparing the values of G’_1_ and η*_1_, regardless of the temperature. A decrease in both parameters with the increase in pH took place until reaching a minimum at pH 5–6.5. From that point on, they increased again at alkaline pH. This behaviour is based on the dependence of protein charges on pH. Since the charges at the isoelectric point were neutralised, the hydrogel formation and stabilization were not ionically favoured and, for this reason, the microstructure was weakened, so the properties obtained were lower. Nevertheless, the viscosity values obtained were lower than those obtained by other authors concerning collagen-based hydrogels. For example, this is the case of the study carried out by Brazdaru et al. on collagen hydrogels containing tannic acid for topical applications [58]. However, according to the results recently reported by Cheng et al., all the systems included in the present study show suitable rheological properties for their injection in the patients [59].

Regarding the tan δ_1_ values, the systems at 20 °C present values lower than 1, which is typical of solid character systems, corroborating what was observed in the frequency sweeps (G’ values above G’’ values). However, the systems elaborated at low temperature (4 °C) show even stronger dependence on pH. Thus, the use of very acidic pH (pH 3) improved the elastic character of the hydrogel (lower value of tan δ). In addition, the values obtained at pH 5 and 6.5 should be pointed out for being the only ones greater than 1 (viscous character).

It is important to point out the effect of pH on protein-based scaffolds (specifically for collagen and gelatin). At pH values higher than the isoelectric point of the protein, the charges produced along the protein backbone favour the formation of hydrogen bonds that may affect the properties of the hydrogel, with a greater effect in the mechanical properties and the biocompatibility of the protein-based hydrogel. Indeed, it has been reported that collagen/gelatin with an isoelectric point between 4 and 5.5 presents better biocompatibility at physiological pH [60].

Based on the results obtained, the use of acidic pH (pH 3) at low gelation temperature (4 °C) or basic pH (pH 10) at higher gelation temperature (20 °C) were presented as the best option for obtaining collagen hydrogels.

#### 3.2.3. Hydrogels Selected

**Figure 5:**
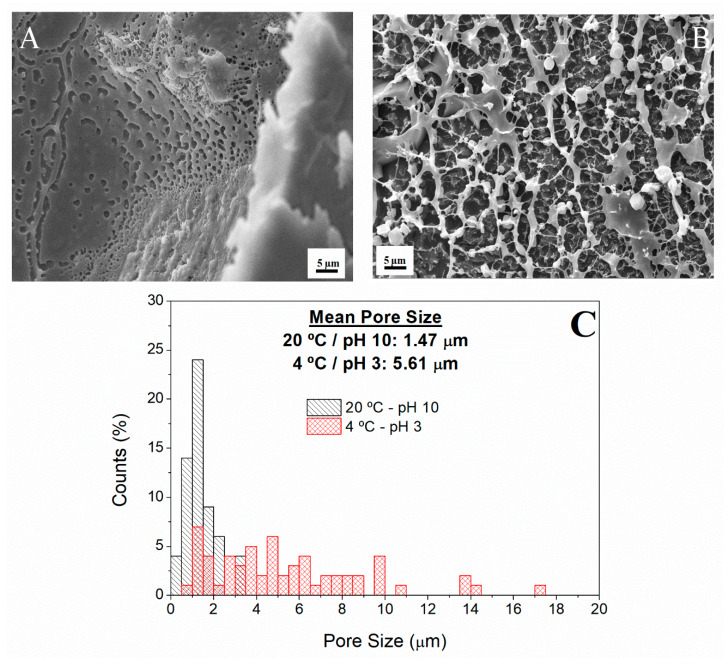
Cryo-SEM images of the hydrogels studied: (**A**) pH 10 at 20 °C; (**B**) pH 3 at 4 °C and (**C)** pore size distribution of both hydrogels produced with the selected conditions.

Further investigation was carried out to compare the microstructure of two of the hydrogels produced using the parameters chosen in the previous sections. The systems selected were those showing the best properties among the hydrogels obtained. It is on this account that this investigation included a morphological characterization of the hydrogel via Cryo-SEM of the hydrogel prepared at 20 °C and pH 10 (Figure 5A) and the hydrogel obtained at 4 °C and pH 3 (Figure 5B). 

Thus, the internal network and the pore size distribution of each of these two scaffolds were analysed to determine whether the structure formed could mimic the extracellular matrix (ECM). The pore size distributions of both scaffolds are also presented in Figure 5C, each one showing a profile that depends on the processing conditions of the hydrogel. The hydrogel prepared at 20 °C and alkaline pH shows a profile with most pores being present in the range between 0.4 and 4.1 μm (black-left dashed columns), whereas the pore size for the hydrogel prepared at 4 °C and acidic pH mainly ranged from 0.9 to 17.1 μm (red-multi dashed columns). In addition, the microstructural image obtained for these scaffolds (Figure 5A,B) revealed a porous microstructure formed by several pores of different sizes, which was in accordance with the results of the pore size distribution. The values obtained are lower than those obtained by Yannas et al. (2013) [61], but similar in values and appearance to the ones obtained by Mousavi et al. (2019) who studied hydrogels based on combinations of chitosan and collagen [62].

The differences observed in the microstructure are due to the differences occurred during the gelation process caused by using different pH values. Acidic pH values encouraged the formation of a less branched gel and, thus, a macroporous structure, whereas the use of basic pH values promoted the formation of a porous structure with a lower pore size [63]. Nevertheless, both structures presented pores throughout the entire sample, which could be favourable to allow efficient nutrient supply to cells [64].

## 4. Conclusions

As a general conclusion, it was possible to develop a gelatinised collagen protein matrix through a gelation process that allows the formation of hydrogels. Several different systems were studied, some of them presenting suitable properties for their potential application in tissue engineering.

The characterization of the raw material revealed a type I collagen protein isolate, with ca. 91 wt % of protein content, an important denaturation degree and an isoelectric point in the range of pH 5–6.

The results obtained indicate that rheological measurements can be regarded as a useful tool to monitor and control the scaffold fabrication process. The optimization of the processing parameters of the collagen-based hydrogels revealed that the most suitable conditions are a gelation time of 2 h, and pH 3 (at 4 °C) or pH 10 (at 20 °C).

The microstructural characterization of the selected systems showed that both hydrogels presented a porous structure that could mimic the ECM. Specifically, the hydrogel formed at basic pH showed a macroporous structure with a pore size range between 0.35 and 4.11 µm, whereas the hydrogel prepared at acidic pH presented a heterogeneous porous structure with a pore size range of 0.88–17.14 µm.

Further studies will encompass the biological evaluation of the most suitable system in order to analyse their suitability to harbour cells and, consequently, to develop tissues.

## Figures and Tables

**Table 1:** G’, tan δ and η* at 1 Hz (G’_1_, tan δ_1_ and η*_1_) and critical strain of the hydrogels as a function of the gelation time (1, 2 and 4 h).

Gelation Time	γ_c_ (-)	G’_1_ (Pa)	tan δ_1_ (-)	η*_1_ (Pa·s)
1 h	0.48 ± 0.08 ^I^	0.53 ± 0.12 ^a^	0.52 ± 0.01 ^A^	0.27 ± 0.06 ^α^
2 h	1.01 ± 0.05 ^II^	1.58 ± 0.42 ^b^	0.15 ± 0.03 ^B^	0.85 ± 0.26 ^β^
4 h	1.01 ± 0.11 ^II^	2.55 ± 0.97 ^b^	0.14 ± 0.04 ^B^	1.74 ± 0.81 ^β^

^I, II, a, b, A, B, α, β^Values with different letters are significantly different.

**Table 2:** G’, tan δ and η* at 1 Hz (G’1, tan δ1 and η*1) and critical strain of the hydrogels as a function of the pH (3, 5, 6.5, 8 and 10) and the gelation temperature (4 and 20 °C).

Temperature	pH	γ_c_ (-)	G’_1_ (Pa)	tan δ_1_ (-)	η*_1_ (Pa·s)
4 °C	3	1.01 ± 0.05 ^I^	1.58 ± 0.42 ^a^	0.15 ± 0.03 ^A^	0.85 ± 0.26 ^α^
	5	1.01 ± 0.20 ^I^	0.22 ± 0.05 ^b^	2.69 ± 0.33 ^B^	0.25 ± 0.08 ^β^
	6.5	0.69 ± 0.10 ^II^	0.01 ± 0.01 ^c^	37.24 ± 3.09 ^C^	0.21 ± 0.03 ^β^
	8	1.01 ± 0.01 ^I^	0.78 ± 0.33 ^d^	0.62 ± 0.12 ^D^	0.52 ± 0.07 ^γ^
	10	1.01 ± 0.03 ^I^	0.82 ± 0.16 ^d^	0.68 ± 0.14 ^D^	0.34 ± 0.01 ^δ^
20 °C	3	0.42 ± 0.08 ^III^	1.01 ± 0.36 ^ad^	0.36 ± 0.05 ^E^	0.57 ± 0.17 ^γ^
	5	0.48 ± 0.11 ^III^	0.29 ± 0.04 ^b^	0.75 ± 0.24 ^D^	0.15 ± 0.03 ^β^
	6.5	0.27 ± 0.05 ^IV^	0.55 ± 0.15 ^d^	0.58 ± 0.10 ^D^	0.31 ± 0.14 ^β^
	8	1.03 ± 0.12 ^I^	1.52 ± 0.45 ^a^	0.22 ± 0.02 ^F^	0.90 ± 0.25 ^α^
	10	1.01 ± 0.21 ^I^	1.38 ± 0.53 ^ad^	0.23 ± 0.04 ^F^	0.85 ± 0.19 ^α^

^I, II, III, IV, a, b, c, d, ad, A, B, C, D, E, F, α, β, γ, δ^ Different letters were included to indicate significant differences between the values obtained.
